# Books

**Published:** 1881-10

**Authors:** 


### Books.

A TREATISE ON THE CONTINUED FEVERS. By James C. Wilson, M.D., with an introduction by J. M. DaCosta, M.D. New York: William Wood & Co. 1881. Wood's Library of Standard Medical Authors.

This book opens with a somewhat desultory introduction, by Professor DaCosta, on the management of fever. "For what," says he, "is the study of its causation, what the care of its discrimination, what the close pursuit of the lesions in the solids and blood, unless we are thus led to a more clearly conceived, more thoughtful, more successful management of the fever?" The writer asserts that good nursing is essential. That cleanliness, cheerfulness and regularity are the three great qualities needed in the sick room. Equally important is ventilation—light and air. Broths, milk and farinaceous food should form the staple articles of diet. He believes in feeding fevers, but thinks there is a tendency to over feed in the earlier stages. "From the cruel practice of refusing water to fever stricken patients, there has been, we all know, a strong reaction." A liberal supply of pure water should be allowed. It quenches thirst, and stimulates the activity of the skin and kidneys. Stimulants are indicated by a failing circulation as denoted by the feebleness of the pulse and faintness of the first sound of the heart. Tremor and delirium are other signs which call for stimulants, as they are mostly—the latter not always—the result of failing nervous power. The most potent means of reducing high temperature is the cold bath, but it is very rarely practicable in private practice. Sponging the skin with cold water, quinine in large doses, and salicylate of sodium are more available. To control the circulation, alcohol is useful by steadying the heart in cases of deficient cardiac action. Quinine and strychnia, in small doses, help. In the "ardent" forms nothing equals aconite. Digitalis is disappointing. Veratrum viride is not mentioned by the writer, and no reference is made to opium. Lastly, the patient should not be forgotten, in the pursuit of the disease.

The seven chapters in the book treat, respectively, of simple continued fever, influenza, cerebro-spinal fever, enteric or typhoid fever, typhus fever, relapsing fever and dengue.

The author says that it has been his aim to describe these familiar diseases with "greater fullness than is usual in the text books, yet without the extreme elaboration that mars the usefulness of some of the special treatises." We think the author, while not attempting anything new, has presented the subjects of which he treats in a compact, yet comprehensive form, and that his object, as expressed in the preface, has been attained. The treatise will be found interesting and useful.

---

A MEDICAL FORMULARY based on the United States and British Pharmacopœias, together with Numerous French, German, and Unofficinal Preparations. By Laurence Johnson, A.M., M.D. New York: William Wood & Co. 1881. Wood's Library of Standard Medical Authors.

The author has attempted to collect in a convenient form, for ready reference, the drugs and preparations in common use, together with formulæ illustrating the manner in which they are combined by good practitioners.

While confining himself as closely as possible to the pharmacopœias, the author has found it necessary to include some unofficinal drugs and preparations which have come into general use since the latest editions of those works In the book, of four hundred pages, he does not include all of the officinal preparations, some of the less important being omitted. The work has evidently been executed with care, and the concise reference to the therapeutic properties of the various drugs discussed, the addition of formulæ in use in a number of hospitals and by private practitioners, the alphabetical arrangement of the contents and a copious index combine to make the book convenient and valuable for reference.

---

Clinical Lectures on the Diseases of old Age. By J. M. Charcot, M.D. Translated by Leigh H. Hunt, B. Sc., M.D., with additional lectures by Alfred L. Loomis, M.D. New York: William Wood & Co. Woods Library of Standard Medical Authors.

The distinguished character of the authors lends, at once, a special interest to this volume.

The contents consists of thirty-one lectures—twenty-one of which were delivered by Professor Charcot. They respectively treat of the general characteristics of senile pathology; the febrile state in the aged; nodular rheumatism and gout, pathological blood-conditions of gout; pathological anatomy of gout; pathological anatomy of visceral gout; semeiology of gout—uric acid diathesis—acute gout—chronic gout; symptomatology of visceral gout; concomitant diseases of gout; etiology of gout; pathology of gout; chronic articular rheumatism and its anatomical lesions; comparison between chronic articular rheumatism and the other constitutional arthropathies, from an anatomical stand point; acute articular rheumatism considered especially in its relations with chronic articular rheumatism and gout; visceral affections in acute and chronic articular rheumatism; symtomatology of chronic progressive articular rheumatism; symptomatology of partial chronic rheumatism and of Heberdens nodosities; etiology of articular rheumatism; treatment of gout and chronic articular rheumatism.

Lectures nineteen, twenty and twenty one are devoted to the consideration of the clinical importance of thermometry in old age. The remaining lectures, by Professor Loomis, treat of senile pneumonia; senile chronic catarrh of the bronchi; asthma; atheroma—fatty heart; cerebral hemorrhage—apoplexy; cerebral softening, chronic gastric catarrh; senile constipation; senile hypertrophy of the prostate gland.

Of the remedies indorsed by Professor Charcot, in the treatment of gout, colchicum is ranked first. He compares its action, in cases where it is well borne, administered in small doses, to quinine in intermittent fever. It causes the inflammation and terrible gouty pain to disappear as if by magic. It should be cautiously used. In acute and chronic rheumatism the drug is utterly useless. Quinine, salts of potassium and lithium, hyoscyamus, opium and blisters, the size of a twenty-cents coin, are recommended. Cold to the joints is dangerous, and the use of leeches is now abandoned. In the management of chronic articular rheumatism he enumerates alkalies, iodine, arsenic, guaiacum etc., and adds "we must acknowledge that chronic rheumatism is a disease which, in the majority of cases, baffles all the resources of medicine." Altogether, it is an entertaining and trust-worthy treatise upon an interesting theme.

---

The Mothers Guide in the management and feeding of Infants. By John M. Keating, M.D. Philadelphia: Henry C. Lea's Son & Co., 1881.

This little volume was opened with genuine pleasure. The subject being of transcendental importance to the human race we had hoped to find its pages filled with words of wisdom. How grievously we were disappointed we shall allow a few extracts to attest. The author claims that he was prompted to write the book by the urgent solicitations of some young mothers who have placed themselves and their children under his guidance. It is written for mothers; not exclusively for the professional reader. Let us see how he advises them. Part first is devoted to general management from birth to dentition.

On page 28. He says: "The principle qualification—[of the milk] I was about to say the only one of importance—is that it should be *fresh.*"

Page 33. "In very young infants, who are from the first bottle fed, the food should consist simply of milk diluted with about half water, and a small quantity of white sugar. . . . . For an infant of the age about which we have just been speaking [under third or fourth month] add a pint of *boiling* water to a pint of milk, put in a small pinch of table salt, slightly sweeten with pure loaf sugar and strain. . . . A couple of inches square of Coopers sheet gelatine, previously soaked in cold water, can be added to the boiled water."

Page 37. "I have said, only a few pages back, that the only true food for infants was milk, and urged the importance of avoiding farinaceous food as a diet. I said this advisedly, because we so often hear of the attempts at feeding babes with corn starch and such like, with such bad results. . . . At the same time I will make a statement that will be an apparent contradiction, which is that it is necessary, after a child is a few weeks old, to add one of these very substances, indigestible in itself, in small quantities, to make the food easier of digestion, just as the ostrich eats stones, and small birds sand, for the purpose of trituration."

Wonderful to tell! Because, forsooth, a fowl needs sand in its gizzard to grind its food, a child—heaven save the mark—requires flour in its *gizzard* to grind its milk!

He continues: "Arrowroot, barley-water, or oat meal water, and, in fact, various other substances of the kind will be useful for this purpose. . . . In most cases the choice of food is an experiment; with one child barley food will agree best; with another the oat meal; with some the cracked wheat, or prepared wheat."

Our author then proceeds to indorse some half-dozen different brands of so-called "infant's foods." He says: "They will agree well when used to slightly thicken the milk, and in others the various well-known preparations of the cereals, as prepared wheat, farina and such like, will do better."

"For young infants, those between two and five months, the bottle food should be of the consistency of cream; after that age it is better to make it thicker until it resembles gruel."

Page 68. "I am opposed to giving meat juice or broths to infants of such tender age, unless they are specially ordered by the doctor. If your child is ill enough to need them, the doctor should be called at once."

Part second covers the period of dentition, and on page 81 we read, "If the child is ill enough to need medicine, it is time to send for the doctor." Very well; but he adds on page 82, when you are at a distance from the doctor it is well to have several needful articles. The following is his list of harmless remedies to be administered at the discretion of the young and inexperienced mother:

One-grain bromide of potassium powders, powdered alum, one-grain calomel powders, milk of assafœtida, lime-water, bicarbonate of soda, soda-mint, mustard, castor oil, rhubarb in lump, spiced syrup of rhubarb, essence of peppermint, magnesia, syrup of ipecac, syrup of squills, syrup of lactucarium, vaseline, camphor water, elixir of valerianate of ammonia, sweet spirits of nitre, spirit of mindererus, chloral liniment, a good stock of some of the "foods," and tincture of aconite root!

We have searched in vain through the pages of this little book for an earnest, consistent and sustained protest against the too prevalent practice of using farinaceous food for young children, bottle-fed or otherwise, on the ground that it is, for them, indigestible and harmful. We have failed to find among its contents minute directions for making an acceptable substitute for human milk from the milk of the cow, either as regards the qualities of the milk to be selected, or the varying degrees of dilution to which it should be subjected for the purpose of regulating the proportion of casein, butter and sugar to suit the different ages and the different degrees of physical development, or the varying physical conditions of the children for whom it may be prepared. But we forbear. Mixed with some advice that is good, there is much that is fatally fallacious. It is to be hoped that few only will follow this "guide," and that a merciful Providence will shield them from the legitimate consequences of their error.

---

MEDICAL ELECTRICITY; A Practical Treatise on the Applications of Electricity to Medicine and Surgery. By Roberts Bartholow, A.M., M.D., LL.D. Philadelphia: Henry C. Lea's Son & Co. 1881.

The author was prompted to prepare this book for medical practitioners and medical students because his experience in his annual courses of lectures on materia medica and thereapeutics impressed upon him the necessity for such a book.

Of the many excellent works on this subject, some, he thinks, are too voluminous, some too scientific, others are wanting in fullness and accuracy In the preparation of this work he has sought to avoid these errors, and to make an exposition of electricity for remedial purposes, as a medical practitioner, for other medical practitioners.

The six parts into which the contents are divided relate respectively to electro-physics, electro-physiology, electro-diagnosis, electro-thereapeutics, electricity in surgery, and thermo-electricity. In the chapter devoted to the thereapeutic uses of electricity we find the following statements: "No fact in regard to galvanism is more conspicuous than its power to allay spasm." "There is no fact more certain than the power of galvanism to relieve pain." "In neuralgia of the fifth nerve, tic douloureux, or simple neuralgia, the galvanic current affords relief, but is rarely more than palliative." In cervico-brachial neuralgia "I have rarely failed to effect a cure." "In no painful affection is the application of electricity more conspicuous for good than in sciatica." "Lumbago is usually promptly cured by galvanization of the affected muscles." So is myalgia and the soreness and muscular debility following acute rheumatism.

The different kinds of batteries are clearly described the indications for the use of galvanism and faradism are plainly set forth, and the actual and relative value of the various currents fairly discussed. It is a practical treatise, by a practical writer, for practical men. We most cordially recommend it to all who feel an interest in the subject, and this surely includes all intelligent physicians, for none can afford to disregard a remedy of its indisputable value in a large class of troublesome, and some otherwise incurable, affections.

---

Disorders of the Male Sexual Organs. By Samuel W. Gross, A.M., M.D., Philadelphia. Henry C. Lea's Son & Co. Pages 170.

This work treats, in their various forms, of impotence, sterility, spermatorrhœa, and prostatorrhœa. In the first chapter the distinction between impotence and sterility is alluded to, and impotence treated of in the four forms of atonic, psychical, symptomatic and organic.

Atonic impotence, we are informed, depends upon "inflammation and hyperesthesia of the prostatic portion of the urethra, or upon diminished or abolished reflex excitability of the genito-spinal center without the intervention of these lesions." The first class of causes being vastly more frequent. Many illustrative cases are given. Diagnosis is made by the bulbous, or acorn-pointed, bougie. In the majority of cases stricture is present. We find that the author claims that stricture is frequently produced by masturbation. We cannot believe that this habit is so frequently causative of such lesion. A true stricture, we believe, is the product of inflammation; and masturbation rarely can set up the inflammatory process. In the treatment the sound plays an important part. A redundant prepuce, chronic localized congestion, or other apparent cause must be properly treated; in short, the indication is, to relieve the hyperesthesia of the prostatic urethra. Gelsemium, veratrum, bromide of potassium, monobromide of camphor, atropia, laxatives, baths, etc., are recommended in appropriate cases. Galvanism is also treated of.

Psychical impotence is treated of more briefly. As the patient is impotent only because he believes he is, a careful management is instituted in order that he be undeceived.

Symptomatic impotence arises from the use of various medicines, or from injuries of the cerebro-spinal axis.

Organic impotence is that form in which copulation is impossible on account of malformation, injuries, or defects of the penis or other sexual organs. We here find the astounding declaration that the loss of the penis, through disease or design, is irremediable. It would really seem more desirable that so valued an organ should have the power of sprouting from the stump.

The subject of sterility, in persons who are potent, is well and elaborately handled. Azoospermism, aspermatism, and mis-emission, comprise the divisions of the subject. The many varieties and causes of each of these conditions are carefully given. The author claims that in cases of unfruitful couples the husband is at fault more frequently than is generally supposed. He gives one hundred and ninety-two cases which he examined, and of which there were thirty-three (17 per cent.) wherein the husband was the sterile party.

The treatment of these various conditions, the author states, are, for the most part, unsatisfactory, except there be a remediable lesion.

The third chapter is devoted to spermatorrhœa. Of this there are given three varieties: Nocturnal emissions, marked by dream or sensation; diurnal pollutions, excited by mechanical or psychical cause; spermatorrhœa, in strict sense of the term, the emission occurring without erection or sensation, or during urination or defecation. The term spermorrhagia is set down especially for this third division. Several causes are assigned for this (or these) affections; "by far the most common are hyperesthesia and chronic inflammation of the prostatic portion of the urethra, which are generally induced by masturbation; and these morbid conditions are just as important in its production as they are in the causation of impotence."

In diagnosis, the microscope very properly is offered as an invaluable means of determining if the fluid emitted is semen. The prognosis is not made so unfavorable as it is by many writers. With this wiew we most heartily agree. The usual hygienic rules are impressed for the treatment. All lesions or functions of the sexual or neighboring organs, which may excite or prolong the disease, must receive attention. The irritability of the prostatic urethra must be relieved, as pointed out in the treatment of one of the forms of impotence. Stricture, on which may depend either this disease or impotence, must have proper treatment. Prof. Gross, in speaking of remedies here applicable, sums up: "The steel bougie, bromide of potassium and atropia, are especially adapted to cases of nocturnal emissions; and electricity, ergot and strychnia, are the most reliable agents in diurnal pollution and spermorrhagia."

In the fourth and last chapter, we have nine pages devoted to prostatorrhœa, "an excessive secretion of a clear, viscous fluid, dependent upon chronic catarrhal inflammation of the tubular glands of the prostate." The etiology, clinical history, pathological characters, diagnosis, prognosis and treatment are very well set forth. It is correctly pronounced a most obstinate affection, but usually amenable to persistent treatment. The greatest injury it may inflict seems to be the resulting mental anxiety. Among the remedies given, we mention "the warm hip-bath; bougie; bromide of potassium and atropia, combined with tincture of hyoscyamus and bicarbonate of potassium if there be vesical irritability, etc.; ergot; sitz-baths; the injection of thirty grains of nitrate of silver to the ounce; and the application of flying blisters to the perineum."

Throughout the work we find "gland of the penis" often substituted for glans penis. We see no need for such translation. It is not more elegant, and is less distinctive than the term in common use.

On the whole, the work is a valuable one. We must object somewhat to the definition and classification of spermatorrhœa. But the book, properly studied, will accomplish vast good by upsetting some of the teachings of the multitude of careless, ignorant, or designing writers who have gone before.

---

Anatomical Studies upon Brains of Criminals. By Moriz Benedict, Vienna. Translated by E. P. Fowler, M.D., New York. New York, Wm. Wood & Co. Pages 185.

This work is the labor of one who has evidently given much time and investigation to the subject of which it treats. We find fifteen pages devoted to the introduction, giving a most minute description of the numberless fissures and lobes that go to make up what the author regards the normal type of brain. In complicated description, the ordinary text-books are far outstripped.

After this, we have one hundred and five pages devoted to twenty-two "observations"—that is to the records of the post-mortem examination of the brains of as many criminals. In these, the fissures and convolutions aforesaid are compared to those the author has fixed as the normal type. The remaining pages are devoted to "recapitulations," or deductions from the observations.

We are not satisfied that there exists, or that any one knows to exist, any type so definite as is here required for a starting point. The descriptions and "observations" are very complicated, even, we believe, to an expert. It is not proven, in these autopsies, that the deeds or mental peculiarities, of these criminals, were assignable to slight variation from this arbitrary normal type of brain. The author is even more self confident than the enthusiastic phrenologists.

We quote from recapitulation 11: "For many of the descriptive details here given, such as are absent in all previous cerebral representations, we are indebted to the special attention which I have bestowed upon these brain-specimens. This keen observation I owe to a casuistic principle heretofore too much neglected by cerebral-anatomists." Let us, at least, commend the author's modesty.

Again (page 158): "It is self-evident that the observations here collected are the result of an *a priori* conviction that the constitutional (eigentliche) criminal is a burdened (belastetes) individual; that he has the same relation to crime as his next blood kin, the epileptic, and his cousin the idiot, have to their encephalopathic condition (and its results. Tr)." This may prove interesting to a criminal lawyer (for the defense); but, for our part, we still believe that most criminals are more criminal than insane. At any rate, though, there is the consolation that we must kill the accused, and examine his brain, to satisfy ourselves that his peculiar and remarkable fissures and convolutions were inherited from a few generations back.

We cannot restrain ourselves from quoting the last paragraph in the book, even if there be found therein, toward its close, the secret of our failing to appreciate the value of the author's labors: "If haply in one of these institutions some future professor—somewhat mocking Lombroso—exclaims to his pupils. 'The heads of criminals must not be too large or too small, not too broad or too narrow, not too high and not too low, what then should they be?' The proper reply would at once echo back: 'yes, indeed, these heads may be too large or too small, too narrow, or too broad, or too high or too low; they may be atypic. For atypy predisposes to mental disturbance, to epilepsy and to psycho-physical abnormalities of all sorts; or it may be significant of cerebral disease. But for the criminalist it is necessary that his head should not be hollow, in order that he may be able to follow investigations originating from other sources than himself; and in order that he may not be incited to slander other investigators because he himself is incapable of grasping principles, it is necessary that his head should not be evil." Such petulance might lead one to suspect that the author's brain is somewhat fissured (cracked).

The book is handsomely bound, and the translation, in the main, well done.

